# Medicinal Chemistry Aspects of Thiazole and Thiazolidine Derivatives as Fundamental Building Blocks to Design New NNRTIs Against HIV‐1 Reverse Transcriptase

**DOI:** 10.1002/cmdc.70494

**Published:** 2026-09-30

**Authors:** José Arion da Silva Moura, Matheus Vinicius Guimarães de Melo, Diane Regis Santos do Nascimento, Thaynara Paula Warren Bezerra, Mathieu Métifiot, Patricia Recordon‐Pinson, Maira Galdino da Rocha Pitta, Marina Galdino da Rocha Pitta, Michelly Cristiny Pereira, Marie‐Line Andreola

**Affiliations:** ^1^ Center of Research in Therapeutic Innovation Suely Galdino (NUPIT SG) Biosciences Center Universidade Federal de Pernambuco (UFPE) Recife Pernambuco Brazil; ^2^ UMR 5234, Microbiologie Fondamentale et Pathogénicité Université de Bordeaux, CNRS Bordeaux France

**Keywords:** antiviral therapy, antivirals, chemistry, drug design, pharmacodynamic, pharmacology

## Abstract

The therapy to treat the Human Immunodeficiency virus (HIV) and decrease the viral load has been a challenge since its discovery due to its ability to mutate, escape from drug therapies, and immunologic system. Consequently, the development of new therapies, especially potential molecules that focus on specific viral mechanisms to block viral replication, became a critical challenge in recent decades. The Reverse Transcriptase (RT) is an RNA‐ and DNA‐dependent DNA polymerase enzyme that is responsible for transcribing viral RNA into double‐stranded DNA. Non‐nucleoside RT Inhibitors perform important role in HIV therapy, and they are responsible for inhibiting RT by blocking its allosteric site, which shows physico‐chemical properties such as high hydrophobicity and key amino acid‐to‐hydrogen bond interactions. For this reason, designing new molecules with innovative heterocycles compatible with these characteristics can provide new potential inhibitors. For example, pentacyclic heterocycles like thiazole and its derivatives like thiazolidinedione, which have physico‐chemical properties that can interact with the allosteric site of RT, can be a new perspective in designing new Non‐nucleoside RT Inhibitors. This work reviews the scientific literature concerning these compounds and can help the design of new antiviral therapeutics.

## Graphical abstract

HIV‐1 reverse transcriptase (HIV‐1 RT) is a polymerase enzyme and a key target in HIV‐1 therapy. The discovery of selective HIV‐1 RT inhibitors has highlighted thiazole scaffolds and their derivatives, thiazolidine‐2‐one and thiazolidine‐2,4‐dione, as promising structures for the rational design of novel non‐nucleoside reverse transcriptase inhibitors (NNRTIs) with potential antiviral activity that may improve future therapeutic strategies.

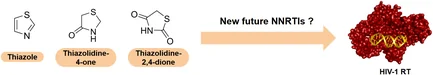

## 1 Introduction

The development of effective therapies to defeat the Human Immunodeficiency virus (HIV), which can cause Acquired Immunodeficiency Syndrome (AIDS), remains a challenge due to the virus’ high mutation rate and resistance capability. Current treatment approaches can be divided into either Pre Exposure Prophylaxis (PreP) or Post Exposure Prophylaxis (PEP), both involving a combination of two or more antivirals that target enzymes of the virus life cycle [1]. Additionally, recent therapeutic strategies have introduced more effective pharmacological options, and in the near future, it is expected that a single‐dose, biannual regimen, as in the case of Lenacapavir, will become available [2].

The anti‐HIV pharmacological classes can be divided into entry inhibitors, protease inhibitors, integrase inhibitors, and reverse transcriptase inhibitors (RTIs) [1]. In fact, RTIs can be divided into two more classes according to their binding sites in different pockets of the RT, blocking the conversion of viral RNA into viral DNA to be integrated into cellular DNA. Nucleoside/Nucleotide Reverse Transcriptase Inhibitors (NRTIs/NtRTIs) bind the catalytic site of the enzyme and act as chain terminators. Non‐Nucleoside Reverse Transcriptase Inhibitors (NNRTIs) are a class of molecules that target an allosteric site of the enzyme, inducing conformational modifications that block the reverse transcription mechanism [3].

Heterocycles are the most important building blocks for designing new molecules. Indeed, NRTIs have, heterocycles, modified purines and pyrimidines to guide molecules toward enzyme’s polymerisation site and mimetize natural nucleic acids. Notable examples include Abacavir [4], Tenofovir [5], and Entecavir [6] as purine derivative structures and Lamivudine [7], Emtricitabine [8], and Zidovudine [9] as pyrimidine derivative structures [3]. On the other hand, NNRTIs present a different strategy to fit inside the allosteric site, presenting heterocycles as pyridine in nevirapine [10], indole in delavirdine [11], benzoxazine in efavirenz [12], triazole in doravirine [13], as well as pyrimidine in rilpivirine [14] and etravirine [15, 16].

The physicochemical properties of these various heterocycles have enabled the development of safe and effective therapeutics that are widely utilized in clinical settings for both the prevention and treatment of the clinical manifestations associated with HIV infection [17, 18]. However, recent studies have demonstrated the increasing resistance rate of the virus to some of these treatments, predominantly resulting from amino acid mutations within the allosteric site of reverse transcriptase (RT) [19, 20]. In this context, thiazole, thiazolidin‐4‐one, and thiazolidine‐2,4‐dione heterocycles have emerged as key structural scaffolds for the synthesis of potential non‐nucleoside reverse transcriptase inhibitors (NNRTIs), as they are capable of interacting with amino acid residues that are essential for the activity of this enzyme.

The thiazole nucleus (Figure 1A) is a promising aromatic five‐membered heterocyclic ring containing both nitrogen and sulfur heteroatoms, and sometimes it can be found condensed with other circles, i.e., benzothiazole (Figure 1B). It is a structural motif found in molecules exhibiting a wide range of pharmacological activities, including anti‐inflammatory [21], antibacterial [22], antifungal [23], anticancer [24], and antiviral [25]. Notably, it is a key component of Ritonavir, an antiretroviral drug that inhibits HIV protease [26]. Given its versatile pharmacological potential, this nucleus has emerged as an important scaffold in the design and development of new therapeutic agents, particularly within the field of antiviral drug discovery.

**FIGURE 1:**
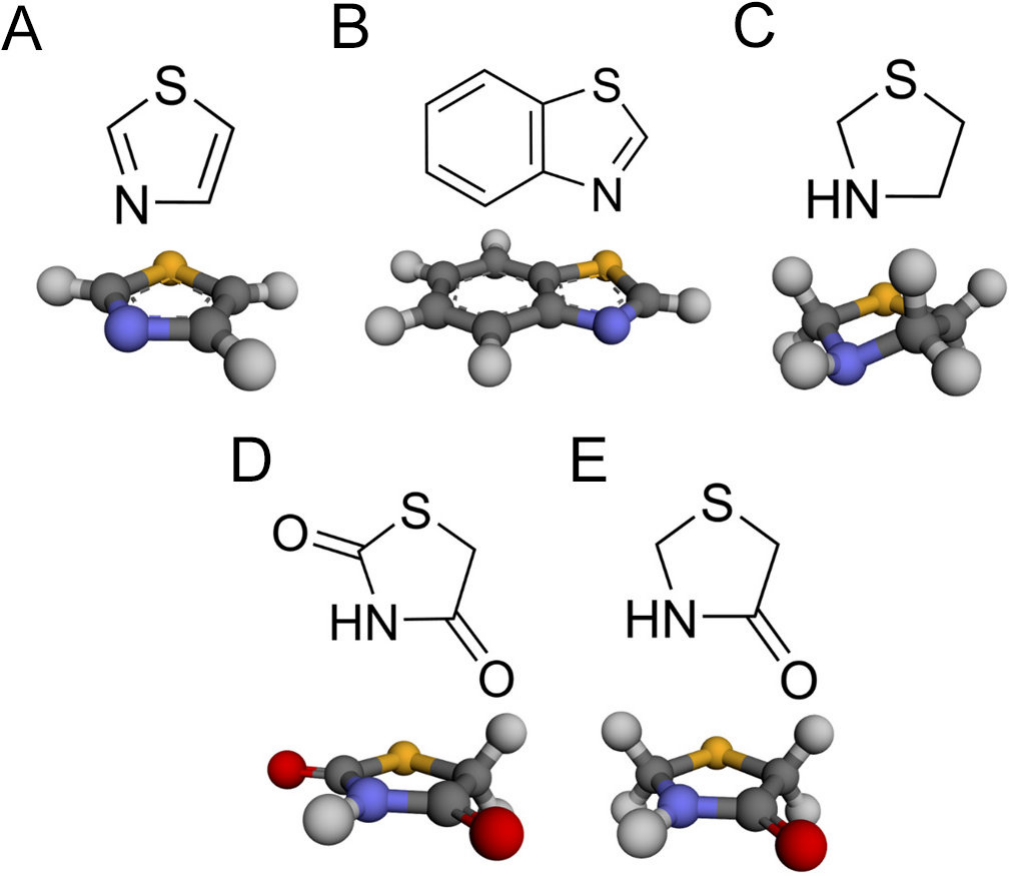
Pentacyclic compounds derived from thiazole (A) in 2D and 3D dimensions, (B) benzothiazole, (C) thiazolidine, (D) thiazolidinedione, and (E) thiazolidine‐4‐one, that can influence the development of new NNRTIs following as scaffolds in the butterfly‐like or horseshoe‐like conformation. Also, the 3D conformation helps in the perspective of designing molecules with rigid structure due their planar characteristics.

Thiazolidine (TZD) (Figure 1C) is a saturated derivative of thiazole (Figure 1A) through the saturation of its double bonds. Thus, thiazolidinedione (Figure 1D) is a heterocycle derived from thiazolidine, which has two carbonyl groups in the position 2 and 4 of the ring. It can also have one carbonyl group in position 4, yielding thiazolidin‐4‐one (Figure 1E). This ring can be found in therapeutic molecules to treat diabetes mellitus, such as pioglitazone, rosiglitazone, troglitazone, and ciglitazone. They can act as peroxisome proliferator‐activated receptor gamma (PPARγ) agonists, modulating different pathways involved in glucose metabolism, adipogenesis, lipid homeostasis, and inflammation [27]. Therefore, due to their intracellular mechanism, treatment with TZDs can lead to off‐target adverse effects, but designing compounds with high specificity to HIV‐1 RT can reduce these events. However, its extensive pharmacological profile enables the repositioning and rational design of TZD‐based compounds for alternative therapeutic applications, including, e.g., the development of new anticancer and radioprotective [28] agents in the treatment of obesity [29], antifibrotic effects [30], antibacterial [31], and antiviral [32, 33] applications. Furthermore, novel TZD‐based hybrid compounds against HIV‐1 RT can work in modulating opportunistic diseases resulting from HIV‐induced immunodeficiency.

From a chemical perspective, these heterocycles exhibit structural versatility that facilitates the development of promising molecules targeting HIV‐1 RT. This property arises from the planar and compact three‐dimensional conformation of these scaffolds, which enables closer intermolecular interactions with amino acid residues that are essential for reverse transcriptase activity, most notably Lys101, Lys103, Tyr181, Tyr188, and Trp229 [34, 35]. Furthermore, the aromatic character of the thiazole ring, combined with its heteroatoms, nitrogen and sulfur, permits both hydrophobic interactions, i.e., van der waals, pi–pi stacked pi‐cation, and hydrogen bonding within the allosteric site of RT, thereby enhancing the potential for highly effective and selective enzymatic inhibition [36]. Regarding the thiazolidine scaffolds, their planarity, coupled with the presence of electronegative atoms such as oxygen and nitrogen, promotes thermodynamically favorable interactions with the amino acids comprising the hydrophobic allosteric pocket of RT [37, 38]. In contrast to currently available therapeutics for HIV treatment, these structural groups can be extensively derivatized, yielding compounds with diverse spatial and geometric conformations [39, 40]. These features impart chemical properties that can promote more effective and selective inhibition of reverse transcriptase. Furthermore, the balance between the hydrophobicity and hydrophilicity of these scaffolds facilitates the design of compounds with an optimal drug‐likeness profile, allowing for the facile optimization of pharmacokinetic parameters such as absorption, distribution, metabolism, and excretion (ADME) [41, 42].

Thiazole and thiazolidine derivatives have demonstrated promising anti‐HIV activity, paving the way for the development of innovative therapies [43, 44, 45]. The inhibition of HIV‐RT effectively disrupts the viral replication cycle, making it a critical target for antiretroviral drug development [46, 47]. Therefore, this study aims to review the structural and functional properties of HIV RT, focusing on the NNRTIs binding site. Furthermore, this work seeks to analyse thiazole and thiazolidine scaffolds as important structures in the design of novel HIV‐RT inhibitors, thereby supporting future studies on design strategies for potential new antiviral NNRTIs drugs.

## 2 HIV Reverse Transcriptase as a Drug Target

The reverse transcriptase enzyme is a RNA‐dependent and DNA‐dependent DNA polymerase (RDDP) encoded by HIV, and it is involved in the conversion of viral RNA molecules into double‐stranded DNA, a crucial step in the viral replication cycle [48]. This enzyme has emerged as an important target for anti‐HIV drug development in recent decades [49]. However, designing new inhibitors requires strategies to reduce the toxicity of RT inhibitors. These molecules affect human enzymes such as mitochondrial DNA polymerase γ (Pol γ) due to their structural similarity for RT. Earlier inhibitors such as stavudine and didanosine present less selectivity to RT, therefore causing more side effects compared to recent inhibitors like zidovudine [50]. Furthermore, its structural and functional properties have been extensively studied to enable the design of novel inhibitors aimed at enhancing pharmacological activity, improving safety profiles, and overcoming viral resistance [51].

NNRTIs constitute one of the main classes of antiretroviral agents currently used in clinical practice for the treatment of HIV‐1 infection. The binding site of these molecules is an allosteric area near the catalytic domain of reverse transcriptase called Non‐nucleoside Inhibitory Binding Pocket (NNIBP) [51]. This binding site is characterized by a hydrophobic pocket located approximately 10 Å away from the enzyme’s active site, where the predominant chemical interactions, among the co‐crystallized ligands and the protein structure are hydrophobic, primarily mediated by Van der Waals forces [52]. NNRTIs are responsible for disrupting the alignment of deoxyribonucleotide triphosphates (dNTPs), inducing conformational changes in HIV‐1 RT, thereby inhibiting its enzymatic function [53].

Several structural characteristics are considered essential in the design of new HIV‐RT inhibitors, including the presence of one aromatic ring, one hydrogen bond acceptor, and two hydrophobic groups [52]. These structural details can enhance a compound’s ability to interact effectively with key amino acid residues, particularly the hydrophobic residues involved in the stability of the allosteric site of the enzyme [52]. Such interactions are crucial for achieving high binding affinity and inhibitory potency against the HIV‐1 RT.

Key amino acid residues within the HIV‐1 RT play a crucial role in mediating target–ligand interactions and represent strategic points for the development of inhibitory compounds. In the crystallographic complex of HIV‐1 RT with Delavirdine (PDB ID: 1KLM) [54, 55] (Figure 2A), the hydrophobic residues Leu100, Phe227, and Trp229 (Figure 1B), located within the enzyme’s hydrophobic binding pocket (Figure 2C,D), are primarily responsible for stabilizing the co‐crystallized ligand through non‐polar interactions [56]. Additionally, the presence of hydrogen‐bond acceptor groups in the inhibitor structure is essential for establishing hydrophilic interactions with residues such as Lys101, Lys103, Tyr318, and Val106 (Figure 2C), which contribute to the overall stability of the ligand–enzyme complex. Notably, Tyr181 and Tyr188 participate in the majority of hydrogen‐bonding interactions (Figure 2D), underscoring their critical role in maintaining complex stability and supporting the pharmacological activity of NNRTIs [56].

**FIGURE 2:**
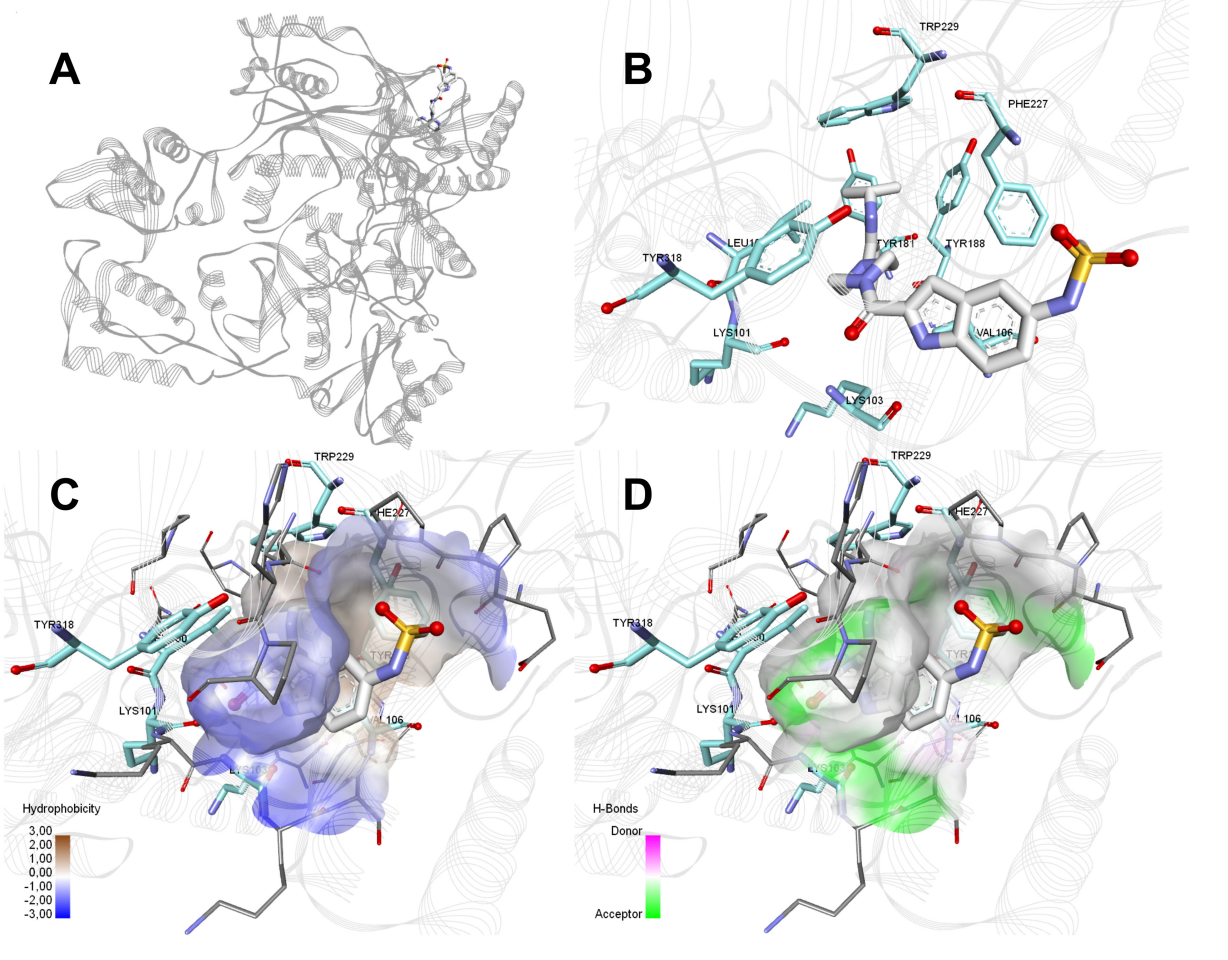
HIV‐1 RT structure (PDB ID 1KLM) in complex to (A) Delavirdine [54, 55]. Three dimensional arrangement of (B) Delavirdine and important amino acids. Surface map of (C) hydrophobicity and (D) H‐bond interactions of HIV‐1 RT inside the NNRTIs allosteric cavity [54, 55].

Furthermore, HIV‐RT is characterized by the presence of two domains, p66 and p51 [47]. The p66 domain was divided into five subdomains. Fingers, palm, thumb, and connection are characteristic of DNA‐dependent RNA polymerase (DdRp) enzymes [57]. In addition, it contains a RNase H domain, with RNase H activity being responsible for the cleavage of the RNA strand within the RNA:DNA heteroduplex replication intermediate [47]. The DNApol catalytic domain is highly conserved, with ASP‐ASP‐GLU (DDE) motifs that are coordinated with two Mg^2+^ or Mn^2+^ ions and by a tertiary structure that is conserved among the Ortervirales [58]. Structural similarity to human RNase H1 can provide secondary events, increasing toxicity [59].

The stability and flexibility of the HIV‐RT heterodimer are critical requirements for its activity. Mutations within the RNAse H domain of HIV‐1 RT have been associated with viral inactivation, leading to the production of non‐infectious virus particles [47]. The RNAse H domain is characterized by the presence of two divalent metals, which represent attractive targets for ligands capable of coordinating metal centers, thereby promoting favorable thermodynamic interactions that result in HIV‐1 RT inactivation. The p51 domain shows the same sequence as the DNA polymerase domain of p66; however, it lacks the RNase H subdomain. It adopts a more compact structure and is deprived of the enzymatic activity. To date, only a limited number of studies have explored molecules capable of interacting with key amino acids within the p51 domain, representing a potential opportunity for novel inhibitor development [47]. Thus, targeting the p51 domain can decrease toxicity and secondary events due to its structural particularities, even when deprived of enzymatic activity, which is important to RT performance [60].

Additionally, amino acid mutations within the allosteric site of RT‐HIV represent a critical global challenge in antiretroviral therapy [61]. These mutations, which involve genetic alterations in the active, catalytic, and allosteric regions of the enzyme, can modify the interaction profiles of various inhibitors. Consequently, certain compounds may lose their binding affinity for the mutated enzyme, resulting in viral resistance and reduced therapeutic efficacy. Several studies have identified key amino acid residues—including Trp229, Phe227, Leu234, and Tyr318—as critical contributors to the binding of NNRTIs within HIV‐RT, playing a pivotal role in preserving enzyme susceptibility to inhibition. These highly conserved residues have been the subject of ongoing investigation aimed at predicting and circumventing potential resistance‐associated mutations [60, 61].

Moreover, the current use of nevirapine has contributed to the emergence of significant mutation in the HIV‐RT structure, resulting in drug resistance in some viral strains [62]. Clinical studies have demonstrated that prolonged use of rilpivirine and etravirine over 48 weeks can lead to the development of viral resistance [63]. Y188C mutant HIV‐1 reverse transcriptase was identified as responsible for nevirapine resistance in vivo and in vitro [64]. Other well‐characterized HIV mutations are K103N, Y181C, and double K103N/Y181C [62]. Recent investigations have explored the antiviral activity of novel dihydrofuro[3,4‐d]pyrimidine derivatives that showed potent antiviral activity against some mutant HIV‐1 strains, such as F227L/V106A and K103N/Y181C, when compared to the standard drugs etravirine and rilpivirine, demonstrating the importance of these amino acids for the development of more effective molecules against mutant HIV‐1 strains. These derivatives have different hydrogen bond donor and acceptor groups in order to stabilize the chemical interactions with some amino acids of hydrophobic pocket, such as Tyr181, Tyr188, Trp229, Lys101, Lys103, and Lys104 [65].

Solvent‐exposed regions of HIV‐1 RT are known regions in enzyme’s structure characterized by polar amino acids that enhance hydrophilic protein‐ligands interactions, as well as improve some drug‐likeness properties [66]. In recent decades, these regions have gained attention due to the focus of extensive investigation to elucidate how their structural characteristics influence drug resistance, selectivity, and binding affinity. Targeting these areas has led to enhanced pharmacological activity in novel diaryl pyrimidine derivatives. Specifically, the incorporation of hydrophilic functional groups into these compounds has improved the hydrogen bond interactions with the solvent‐exposed regions of the enzyme, resulting in increased chemical stability and maintaining favorable RMSD (Root Mean Square Deviation) values below 2 Å in molecular dynamics simulation [67].

Therefore, the structural and chemical characterization of HIV‐RT and its binding domains remains critically important, as it serves as the foundation for the development of potent inhibitors capable of establishing thermodynamically stable interactions and effectively inhibiting the enzyme [65]. Future research should focus on designing molecules with these structures’ parameters, presenting favorable pharmacokinetic and pharmacodynamic profiles and overcoming resistance‐associated limitations [68].

## 3 Role of Thiazole Derivatives in HIV‐1 RT Inhibition

Thiazole and thiazolidine derivatives have demonstrated promising anti‐HIV‐1 activity, contributing to the advancement of innovative therapeutic strategies. Predictions of interactions between HIV‐1 RT and potential inhibitors through molecular docking experiments are commonly reported in studies focusing on the development of new antiviral agents. Hydrophobic interactions involving pi electrons, such as pi–pi stacking and pi–pi T‐shaped are particularly prevalent in the thiazole‐containing compounds due to the inherent aromaticity and electron‐rich nature of the pentacyclic ring system. The thiazole moiety of compound 1 (Figure 3) interacts with Lys223 by pi‐cation and Trp229 by pi–pi interactions (PDB ID 3LP2 [77, 78]). When analysed in a different HIV‐1 RT conformation (PDB ID 2ZD1 [79, 80]), the same compound interacts with the Tyr188 side chain and with Phe227 through a T‐shaped pi interaction. In biochemical assays, compound 1 demonstrated notable inhibition of HIV‐1 RT, with IC_50_ values of 4.5 ± 0.5 µM for RNase H activity and 8.0 ± 0.0 µM for RNA‐dependent DNA polymerase (RDDP) activity [69].

**FIGURE 3:**
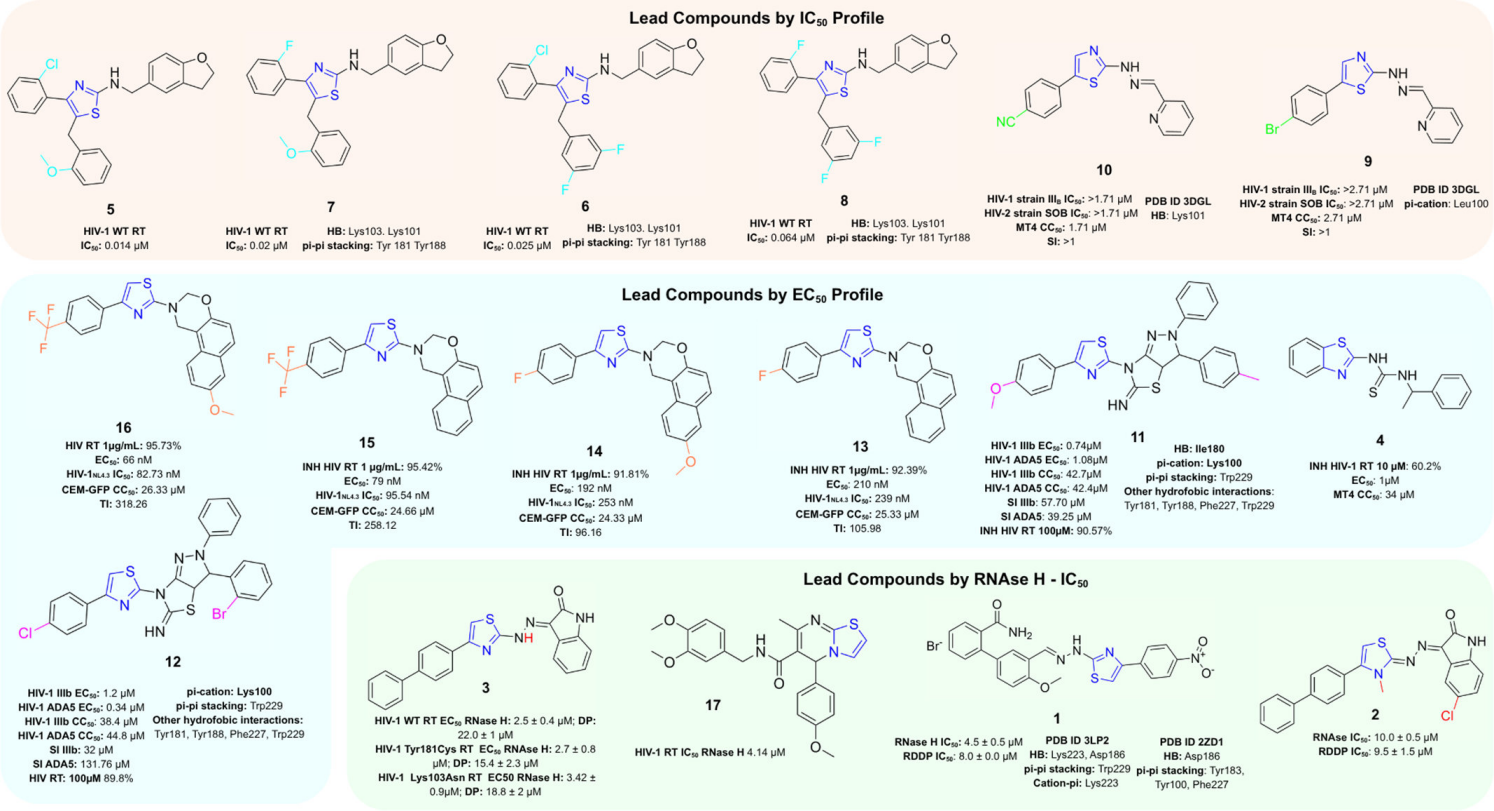
Thiazole derivatives reported as potential molecules against HIV‐1 RT and their biological activity by EC_50_, IC_50_, CC_50_, enzyme inhibitory profile (INH) profiles and in silico interactions [69, 70, 71, 72, 73, 74, 75, 76]. Dark blue highlights the thiazole scaffold, while red, light blue, green, pink, and orange indicate the most effective R substituents within the same series.

Compound 2 (Figure 3), an Isatin‐thiazole hybrid, also performed showed inhibition rates in RNase H (IC_50_ 10.0 ± 0.5 µM) and RDDP (IC_50_ 9.5 ± 1.5 µM) activities [70]. In a previous study, with an analogue molecule, compound 3 (Figure 3), the thiazole ring did not interact directly with the RT, but it was important as a scaffold. Despite the absence of direct binding interactions, this molecule effectively inhibited the HIV‐1 RT in enzymatic assays, wild‐type (EC_50_ RNase H 2.5 ± 0.4; DP 22.0 ± 1) HIV‐1 Tyr181Cys RT (EC_50_ RNAse 2.7 ± 0.8; DP 15.4 ± 2.3) and HIV‐1 Lys103Asn RT (EC_50_ RNase H 3.42 ± 0.9; DP 18.8 ± 2.7) [71]. Additionally, at a concentration of 10 µM, the benzothiazole derivative 4 (Figure 3) inhibited HIV‐1 RT by 60.2%, whereas under the same test conditions, the reference drug efavirenz was inhibited by 96.4% in the enzymatic assay. In vitro, this molecule displayed an EC_50_ of 1 µM against HIV‐1, with moderate cytotoxicity against MT4 cells (CC_50_ = 34 µM) [72].

Trisubstituted thiazo‐triazoles adopted favorable conformations within the NNRTI‐binding site of HIV‐1 RT, where the nitrogen atom from the thiazole ring formed hydrogen bonds with Lys101 and 103. Also, this scaffold proved essential for molecules 5–8 (Figure 3), facilitating pi–pi stacking interactions with Tyr181 and Tyr188. In an enzyme‐based antiviral in vitro assay, these compounds exhibited significantly greater inhibitory potency than nevirapine (NVP), with IC_50_ values ranging from 0.014 to 0.064 µM, compared to 2.1 µM for NVP. Similarly, in cell‐based assays, these compounds demonstrated EC_50_ values around 0.01 µM, surpassing the 0.031 µM value observed for NVP [73]. On the other hand, the thiazole moiety from compound 9 interacted with Leu100 by pi–cation interaction, while compound 10 formed a hydrogen bond with Lys101, contributing to their respective binding affinities within the NNRTI‐binding pocket [74].

The thiazole scaffold is also present in compound 11 (Figure 3), which interacts through hydrophobic bonds with key residues within the HIV‐1 RT NNRTI‐binding site, including Tyr181, Tyr188, Phe227, Trp229, and others. However, the thiazole ring does not participate directly in any of these interactions. In biological assays, compound 11 demonstrated a good enzyme inhibition of 89.80%, while its analogue 12 (Figure 3) achieved 90.57% inhibition at a concentration of 100 μg/mL. In cell‐based assays, both compounds showed promising antiviral activity against HIV‐1 III_B_ with EC_50_ values of 1.08 and 0.74 μg/mL, respectively, and against HIV‐1 ADA5 with EC_50_ values of 1.08 and 0.34 μg/mL, respectively [34]. Additionally, compounds 13–16 (Figure 3) displayed the most potent interaction profiles in terms of inhibitory activity, achieving an IC_50_ of 82.73 ± 2.25 nM [75].

The synthesis and antiviral evaluation of 28 novel thiazol[3,2‐d]pyrimidine compounds revealed, in biological assays, their capacity to inhibit HIV‐1 RNase H activity, with an IC_50_ value of 4.14 μM for compound 17 (Figure 3). These compounds exert their antiviral activity through an allosteric binding mechanism, enhancing interactions with both the p51 and p66 subunits of HIV‐1 reverse transcriptase [76]. This dual engagement contributes to improved inhibitory potency and specificity, offering a promising strategy for enzyme restriction and the development of next‐generation anti‐HIV agents.

## 4 Role of Thiazolidine‐4‐One Derivatives in HIV‐1 RT Inhibition

Based on the butterfly‐like configuration, a series of new 4‐thiazolidinone derivatives hybridized with benzothiazole were developed as potential NNRTIs. Molecular docking studies revealed that the carbonyl group at the C‐4 position of the thiazolidine ring played a critical role in the activity by interacting with Lys101 through a hydrogen bond. An exception was observed with compound 18 (Figure 4), which predominantly established hydrophobic interactions through its benzothiazole ring and the sulfur of the 4‐thiazolidinone moiety. In vitro HIV‐1 RT enzymatic assays demonstrated that compound 18 inhibited HIV‐1 RT by 63% at a concentration of 4 µM, with an IC_50_ value of 0.001 µM. Similarly, compound 19 (Figure 4) achieved 56% inhibition under the same conditions, with an IC_50_value of 0.001 µM. Notably, both compounds exhibited substantially higher potency than the reference drug NVP, which showed an IC_50_value of 0.3 µM [38].

**FIGURE 4:**
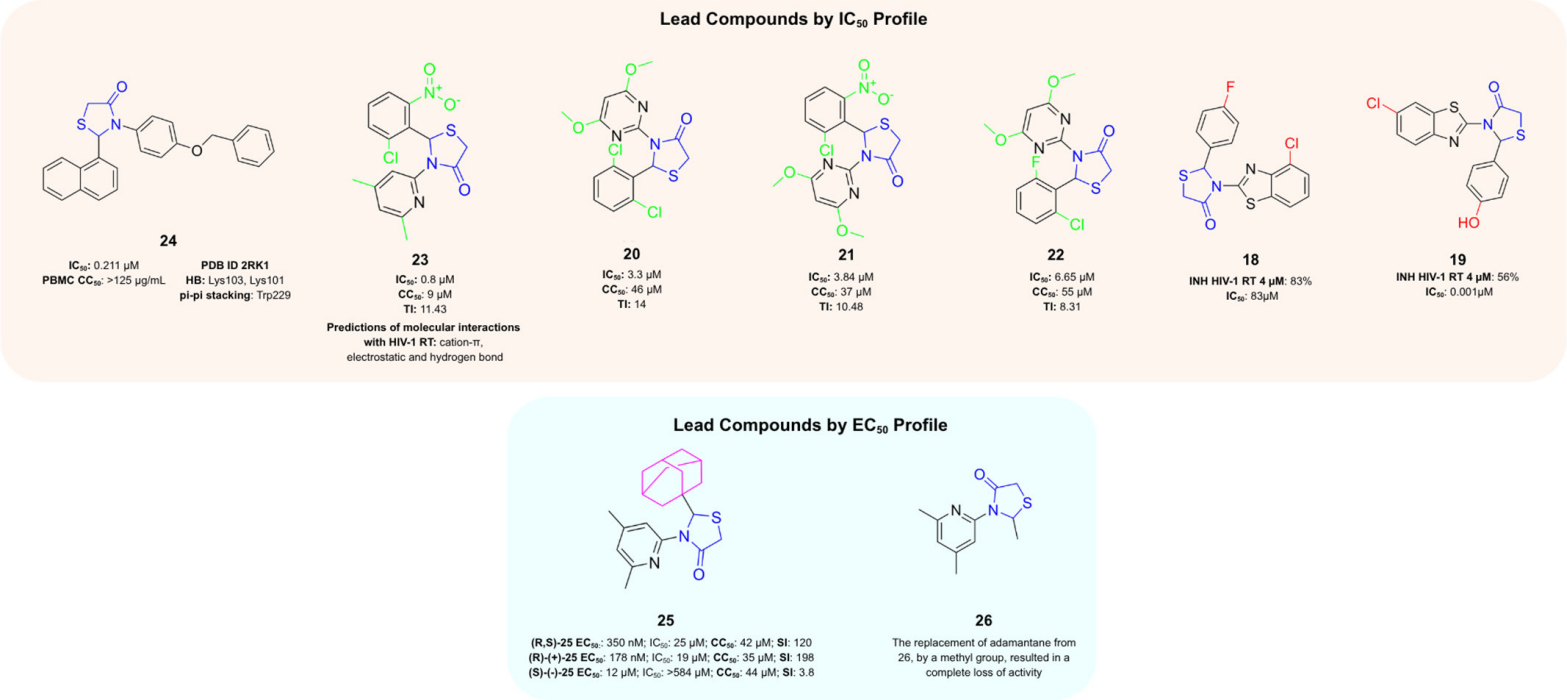
Thiazolide‐4‐one derivatives reported as potential molecules against HIV‐1 RT and their biological activity by EC_50_, IC_50_, CC_50_, enzyme inhibitory profile (INH), and in silico interactions [38, 81–83]. Dark blue highlights the thiazolide‐4‐one scaffold, while red, green, and pink indicate the most effective R substituents within the same series.

A novel series of 4‐thiazolidinone was evaluated against isolated clinical strains exhibiting resistance to first‐line antiretroviral drugs. In addition, their resistance profiles to nevirapine were confirmed. Among the tested compounds, compound 23 (Figure 4) demonstrated the most promising activity with a CC_50_ value of 9 µg/mL, an IC_50_ value of 0.8 µg/mL, and a therapeutic index (TI) of 11.43, as observed for compounds 20–22 (Figure 4). Molecular docking experiments revealed that compound 23 (Figure 4) established pi–cation, electrostatic, and hydrogen bond interactions within the HIV‐1 RT binding site, with the carbonyl group of the thiazolidinone ring acting as a hydrogen bond acceptor [81].

The association between 4‐thiazolidinone and a naphthalene group, characterized by the hydrophobic nature of its fused aromatic rings, showed good inhibitory activity against HIV‐1 RT. In particular, compound 24 (Figure 4) highlighted the critical role of the 4‐carbonyl group in the thiazolidinone ring, which stabilized the ligand through the formation of two hydrogen bonds with Lys101 and Lys103. Additional interactions, including pi–pi stacking with Trp229 and halogen bond with Lys103, were observed between the hydrophobic regions of the molecule and key amino acid residues within the binding pocket. Molecular dynamics simulation supported the thermodynamic stability of this complex at the docking site. In vitro enzymatic assays demonstrated that compound 24 inhibited HIV‐1 RT with an IC_50_ value of 0.211 µM and exhibited low cytotoxicity, with a CC_50_ value greater than 125 µg/mL [82].

The hybridization of the adamantane moiety, as found in amantadine, a clinically used antiviral agent against influenza virus, with the 4‐thiazolidinone scaffold resulted in a series of compounds with notable anti‐HIV activity. Among these, the racemic mixture of 25 (Figure 4) reached an EC_50_ value of 350 nM against HIV‐1. To evaluate the contribution of the adamantane group to the antiviral activity, compound 26 (Figure 4) was produced with a structure identical to that of compound 25, replacing adamantane with a methyl group, which resulted in a complete loss of activity. Furthermore, the enantiomer (R)‐(+) of 25 demonstrated superior efficacy when compared to the racemic mixture, with an EC_50_value of 178 nM against HIV‐1. In enzymatic assays against HIV‐1 RT, the racemic mixture of 25 showed an EC_50_ of 29 µM, whereas the (R)‐(+) of 25 exhibited a lower EC_50_ of 19 µM, confirming the enhanced activity of the enantiomeric form [83].

## 5 Role of 2,4‐Thiazolidinediones Derivatives in HIV‐1 RT Inhibition

The incorporation of two hydrophobic moieties, one comprising a phenylacetamide aliphatic group attached to the nitrogen of the thiazolidine‐2,4‐dione ring and the other an aromatic group at the C‐5 position, led to the development of two novel HIV‐1 reverse transcriptase inhibitors (Figure 5). Compound 27 exhibited 73% inhibition of HIV‐1 RT with an IC_50_ value of 1.31 µM, and compound 28 had 58% inhibition, but with a higher IC_50_ value. Molecular docking studies predicted that the 4‐carbonyl of the thiazolidine‐2.4‐dione ring forms a hydrogen bond to Lys103, while the additional hydrophobic substituents contribute to stabilizing the molecule within the RT allosteric binding pocket [84].

**FIGURE 5:**
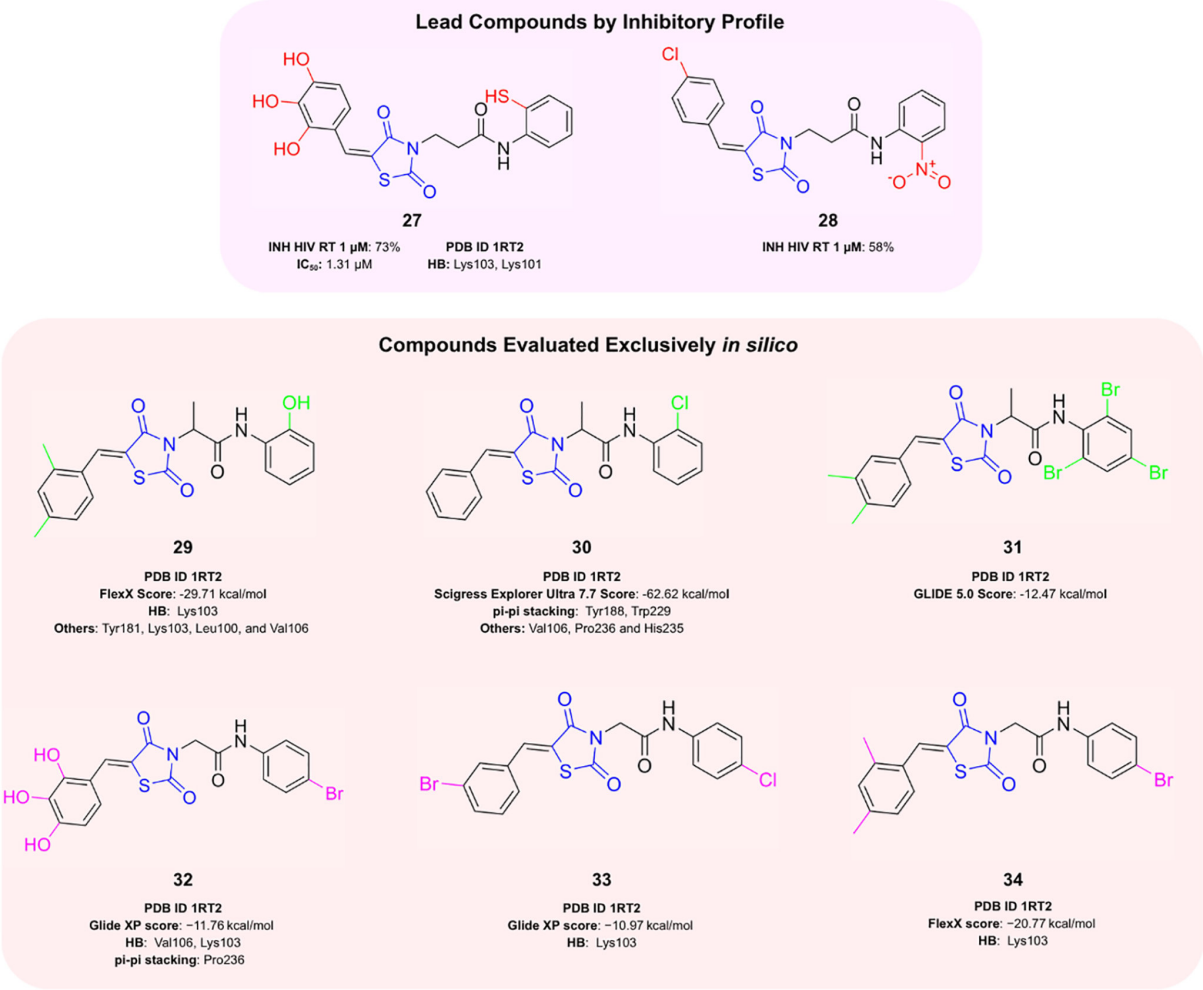
Thiazolidinedione derivatives reported as potential molecules against HIV‐1 RT and their biological activity by IC_50_, enzyme inhibitory profile (INH) and in silico interactions [84, 85, 86]. Dark blue highlights the thiazolidinedione scaffold, while red, green and pink, indicate the most effective R substituents within the same series.

Two hundred eighty thiazolidinedione analogs were evaluated against HIV‐1 RT by virtual screening using three different software platforms, GLIDE 5.0, FlexX 1.0, and Scigress Explorer Ultra 7.7. Key target residues Lys103 and Leu104 displayed stable hydrogen bonds with the ligands. In FlexX simulations, compound 29 (Figure 5) achieved a docking score of −29.71 kcal/mol, with its thiazolidine carbonyl group establishing a strong hydrogen bond with the amino acid group Lys103. Scigress Explorer Ultra 7.7 analysis showed that the thiazolidine ring of compound 30 (Figure 5) (score −62.62 kcal/mol) interacted with the side chain of Val106. Conversely, in GLIDE 5.0 analyses, the thiazolidine ring of the best‐ranked compound 31 (Figure 5) (score −12.47 kcal/mol) did not form any direct interactions with the target molecule [85].

Following the previous study, the same authors analyzed a new series of thirty analogue compounds, modifying the spacer group from methylbutanamide to butanone. Virtual screening using GLIDE identified compound 32 (Figure 5) as the top‐performing molecule, with a docking score of −11.76 kcal/mol, where the carbonyl group of the thiazolidine ring formed a strong hydrogen bond with the terminal amino group of Lys103. A similar interaction was observed for compound 33 (Figure 5), which achieved a score of −10.97 kcal/mol. In FlexX simulations, the carbonyl group of compound 34 (Figure 5) (score: −20.77 kcal/mol) also engaged in a significant hydrogen bond with Lys103. Notably, in Scigress Explorer Ultra 7.7 analyses, the highest‐scoring compound did not establish interactions between the thiazolidine‐2,4‐dione moiety and the target protein [86].

Unfortunately, only a limited number of recent scientific reports have highlighted thiazolidine‐2,4‐dione as a promising scaffold in the design of novel HIV‐1 RT inhibitors. Nevertheless, this gap in the literature presents valuable opportunities for further research and exploration of this molecular class. Considering its potential to interact with key residues such as Lys103 and Leu104, along with its favorable electronic properties and inherent hydrophobicity, which enable its accommodation into the NNRTIs binding pocket of HIV‐1 RT, the thiazolidine‐2,4‐dione derivatives represent a compelling nucleus for the development of novel anti‐HIV‐1 agents.

## 6 Chemistry Aspects in Drug Design of New HIV‐1 RT Inhibitors

Pentacyclic derivative compounds derived from thiazole (Figure 1A), thiazolidine (Figure 1C), and carbonyl‐containing thiazolidines, such as 4‐thiazolidine (Figure 1E) and thiazolidinedione (Figure 1D) structures, are characterized by a three‐dimensional compact arrangement. These scaffolds often incorporate chemical motifs such as benzothiazole (Figure 1B) and other aromatic systems. This polycyclic conformation enhances both steric and electronic interactions with the allosteric site of the HIV‐1 RT enzyme, essential for its replicative function, and can play a role in the NNRTIs characteristic of butterfly‐like or horseshoe‐like conformation [38, 87]. The rigidity of these molecules increases the conformational stability of the ligand‐enzyme complexes, thereby optimizing molecular fitting within the enzyme’s hydrophobic region. Compounds with this structural profile have demonstrated high binding affinity for specific regions of the HIV‐1 RT p66 subunit, including in drug‐resistant mutant strains, due to their favorable chemical geometry and ability to form multiple interactions with key catalytic residues such as Tyr181, Tyr188, and Trp229 [81].

A predominant chemical characteristic of these pentacyclic structures is their hydrophobicity, which is influenced by the presence of multiple fused aromatic systems and lipophilic substituents, such as methyl and aryl groups [88]. In turn, the nonpolar character can help in pharmacokinetics and pharmacodynamics mechanisms like passive diffusion across the cell membrane and insertion into hydrophobic regions of the enzyme, particularly NNIBP. Notably, the introduction of bulky and hydrophobic substituents can enhance the ligand’s affinity for the target, as observed in derivatives containing benzothiazole and isatin cores [38]. Additionally, the presence of heteroatoms, specifically oxygen, nitrogen, and sulfur, within the rings enables the formation of hydrogen bonds with polar residues of the enzyme, such as Lys101 and Lys103, further reinforcing the stability and efficacy of the complex [89].

From an electronic perspective, pentacyclic compounds are characterized by highly conjugated aromatic systems, which confer remarkable thermodynamic stability, making them less susceptible to nonspecific chemical transformations in biological environments. The high stability of pentacyclic aromatic systems, combined with their ability to engage in pi–pi interactions, is crucial for the development of effective HIV‐1 RT inhibitors, including those active against resistant variants [90]. Modifications to peripheral rings, such as halogenation or pi‐system expansion, have been successfully employed to modulate pharmacokinetic properties and enhance affinity against resistant viral strains. Therefore, the rational analysis and exploitation of these electronic and structural properties are fundamental for the optimization of new NNRTI candidates. Thus, pi‐pi interacions contribuite to ligand binding within the HIV‐1 RT binding pocket, particularly through interaction with aromatic redidues [91, 92] .

## 7 Medicinal Chemistry Strategies for New NNRTIs Optimization

Several lead optimization strategies, most notably classical and non‐classical bioisosterism, molecular hybridization, and chiral resolution, have been extensively applied to improve the RT inhibitory activity of derivatives containing thiazole, thiazolidine‐2,4‐dione, and thiazolidin‐4‐one nuclei.

For the thiazole derivatives, bioisosterism and molecular hybridization emerged as the predominant optimization strategies. Bioisosterism is a fundamental medicinal chemistry approach wherein elements, groups, or atoms are replaced by substituents with comparable physicochemical, electronic, and steric properties, often resulting in modulated pharmacological profiles [93]. Conversely, molecular hybridization involves the combination of two distinct pharmacophores with intrinsic biological activities into a single molecule, aiming to achieve a synergistic or enhanced pharmacological effect [94]. Applying this rationale to compounds 2 and 3 (Figure 3), the authors hybridized thiazole and isatin cores to investigate their HIV‐RT inhibitory potential. Compound 2 demonstrated promising activity against RNase H (IC_50_ = 10.0 ± 0.5 µM) and RDDP (IC_50_ = 9.5 ± 1.5 µM) [70]. Subsequent optimization of this prototype via monovalent bioisosterism, specifically, the substitution of a chlorine atom for a hydrogen on the isatin aromatic ring alongside the deletion of the N‐methyl group on the thiazole, afforded compound 3 [71]. This novel derivative exhibited superior inhibitory potency (EC_50_ RNase H = 2.5 ± 0.4; DP = 22.0 ± 1), maintaining strong activity against mutant strains such as HIV‐1 Tyr181Cys RT (EC_50_ RNase H = 2.7 ± 0.8; DP = 15.4 ± 2.3) and HIV‐1 Lys103Asn RT (EC_50_ RNase H = 3.42 ± 0.9; DP = 18.8 ± 2.7) [71]. These findings highlight the robust efficacy of this design strategy, supporting its broader application in the development of potent HIV‐RT inhibitors.

The development of 2,4,5‐trisubstituted thiazole derivatives by Xu et al. yielded prototypes 5, 6, 7, and 8, which displayed RT inhibitory activities surpassing those of nevirapine (Figure 3) [73]. Replacing two fluorine atoms with a methoxy group afforded derivative 5, which exhibited the lowest IC_50_ value of the series (IC_50_ RNase = 0.014 ± 0.0028 µM), underscoring that substituent positioning critically influences biological activity [73]. Notably, the deletion of the methoxy group in compounds 6 and 8 led to a loss in inhibitory potency, validating the essential role of hydrogen‐bond acceptors in the rational design of potent thiazole‐based HIV‐1 RT inhibitors [73]. Furthermore, monovalent bioisosterism strategies were successfully applied to compounds 9 and 10, replacing a bromine with a cyano group, as well as 11 and 12, replacing a methoxy with a chlorine and a methyl with a bromine (Figure 3). Ultimately, these findings illustrate that this optimization methodology is a robust approach for yielding novel lead compounds with profound HIV‐1 RT inhibitory potential and promising prospects for clinical translation [74].

Regarding the thiazolidin‐4‐one derivatives, molecular hybridization again emerged as a prominent strategy. This was primarily achieved by combining the thiazolidin‐4‐one core with various pharmacophores, including benzothiazole, in compounds’ structures of 18 and 19, pyrimidine, in compounds 20, 21, and 22, pyridine, compound 23 and 26, naphthalene, compound 24, or a dual pyridine/adamantane system, compound 25 (Figure 4) [38, 81–83]. Such an approach is exceptionally promising; it imparts structural rigidity to the molecule within the HIV‐RT allosteric binding pocket while enhancing hydrophobic interactions driven by the aromatic rings. Nevertheless, although this methodology is extensively utilized in medicinal chemistry to yield potent lead compounds, it can also inadvertently lead to the generation of derivatives with increased toxicity profiles. To properly assess this liability, in vitro cytotoxicity profiling must be performed on a diverse panel of healthy cell lines capable of accurately reflecting the toxicity of these compounds. Moreover, should cytotoxicity be confirmed for these hybrids, structural optimization strategies should be broadly implemented. Specifically, modifying hydrogen‐bond donor/acceptor moieties, decreasing the overall lipophilicity of the molecule, and replacing halogen atoms are critical steps to mitigate toxicity, thereby improving both selectivity and, consequently, potency.

Aromatic ring substituent modification has similarly been extensively employed. For instance, this approach yielded compounds 20, 21, and 22, highly promising pyrimidine and thiazolidin‐4‐one hybrids active against resistant HIV‐1 strains, displaying IC_50_ values between 0.004 and 0.002 µg/mL [81]. Replacing the two pyrimidine‐bound methoxy to nitro moieties afforded compound 21, which exhibited reduced potency (IC_50_ = 0.003 µg/mL) relative to compound 20 (IC_50_ = 0.002 µg/mL) (Figure 4) [81]. These findings indicate that the methoxy moiety is crucial for maintaining pharmacological activity, presumably by engaging in hydrogen bonding interactions with key amino acid residues within the HIV‐RT NNIBP, Lys101, and Lys103.

For compound 24, an alternative optimization approach commonly employed in medicinal chemistry was utilized: enhancing the molecule’s rigidity and lipophilicity through the addition of a bulky aromatic moiety, in this case, naphthalene (Figure 4) [82]. When combined with the thiazolidin‐4‐one core, this structural modification afforded promising HIV‐1 RT inhibitory activity (IC_50_ = 0.211 µM) [82]. This rationale was similarly applied to compound 25 via the introduction of an adamantane group, a bulky cycloalkane system that promotes favorable van der Waals interactions within the HIV‐RT allosteric binding pocket, resulting in an EC_50_ value of 350 nM against HIV‐1 (Figure 4) [83]. Furthermore, to maximize the pharmacological potency of the prototype, the authors utilized a chiral resolution strategy. Specifically, isolating the (R)‐(+) enantiomer of compound 25 significantly improved the EC_50_ to 178 nM [83]. While these findings validate chiral resolution as a highly effective approach for advancing novel HIV‐1 lead compounds, it is important to acknowledge its inherent limitations. These include technical challenges during isolation, poor synthetic yields, and the risk that the more pharmacologically active enantiomer might concurrently display greater toxicity.

Concerning the thiazolidine‐2,4‐dione moiety, there remains a paucity of studies evaluating the in vitro HIV‐1 antiviral activity of diverse derivatives, as most current literature relies heavily on in silico approaches like molecular docking and molecular dynamics. Even so, rational structural optimization strategies have been applied to advance these derivatives, prominently including the incorporation of hydrogen‐bond acceptors, monovalent bioisosterism, halogen substitution, and the addition of hydrophobic groups. For instance, substituting the hydroxyl group at the 4‐position of the aromatic ring linked to the TZD core with a chlorine atom led to decreased HIV‐1 RT inhibition and an increased IC_50_ value for compound 28 relative to compound 27, which possesses three hydroxyl groups at positions 4, 5, and 6 of the same ring (Figure 5) [84]. These findings corroborate that structural modifications introducing hydrogen‐bond acceptor moieties can significantly potentiate the inhibitory activity of this scaffold. Similarly, for compounds 29–34, modulating the identity and topological placement of halogen substituents on the aromatic ring attached to the acetamide nitrogen afforded compounds with varying binding affinities for reverse transcriptase [85]. Ultimately, these observations indicate that focused modifications of these elements and their substitution patterns provide a strong basis for the rational design of more potent and selective prototypes [86].

In addition to the previously discussed structural optimization strategies, the development of molecules belonging to the thiazole, thiazolidin‐4‐one, and thiazolidine‐2,4‐dione classes has accelerated in recent years due to the widespread application of computational methodologies. This is primarily achieved through the virtual screening of compound libraries using scientifically validated tools, such as molecular docking and molecular dynamics. In this regard, Gao et al. developed a machine‐learning regression model to predict the pIC_50_ bioactivity of 2.547 previously characterized HIV protease inhibitors, aiming to select the most promising compounds for biological evaluation. Consequently, 15 of these molecules demonstrated high potential, exhibiting pIC50 values of at least 7.301. Among these molecules, ritonavir, a well‐known antiviral agent bearing a thiazole scaffold in its structure, stood out. Furthermore, dihydroergotamine mesylate, an alkaloid featuring an indole core as its pharmacophoric group, displayed the best in silico performance, yielding a predicted IC_50_ value of 9.16 nM. These findings demonstrate that combining computational screening strategies with compound optimization methodologies, such as molecular hybridization, can lead to the accelerated development of novel anti‐HIV‐1 agents endowed with enhanced pharmacological activity and greater selectivity [95].

Uslu et al. also developed an AI‐based multi‐stage framework to screen promising molecules against distinct HIV‐1 targets [96]. For this purpose, the authors applied a three‐tier AI‐based workflow encompassing deep learning and molecular docking to accelerate the identification of novel hits, a phase that traditionally spans several years in drug development [96]. The authors utilized a customized Autoencoder‐Long Short‐Term Memory (LSTM) model to generate novel molecular structures that fulfill optimal pharmacokinetic parameters. Subsequently, a geometric deep learning model was deployed to evaluate the potential interactions of these compounds with key HIV‐1 biochemical targets, integrase, protease, and reverse transcriptase, via molecular docking [96]. As a result, the authors successfully identified molecules displaying favorable pharmacokinetic profiles alongside thermodynamically stable binding energies with the molecular targets, particularly against reverse transcriptase. Among these, compounds featuring various heterocyclic cores stood out, most notably, pyridine. These findings underscore the importance of such chemical scaffolds in the development of novel potential HIV‐1 inhibitors. Furthermore, they illustrate how the integration of structural optimization, computational modeling, and synthetic strategies can significantly accelerate the stages comprising the anti‐HIV‐1 drug discovery pipeline.

Nevertheless, the results derived from computational studies must be interpreted with caution. Although these represent robust and scientifically validated methodologies, they are unable to comprehensively replicate complex biological parameters. A notable limitation is that standard molecular docking simulations often assume static and rigid protein models, failing to capture the conformational mobility and dynamic behavior of these targets within a biological environment [97]. Consequently, this can lead to over‐ or underestimated binding affinities, resulting in discrepant biological outcomes [97]. Furthermore, algorithms employed to design molecules with favorable pharmacokinetic and pharmacodynamic profiles against HIV‐1 targets, most notably reverse transcriptase, cannot faithfully predict a compound’s capacity to interact effectively with its target in vivo [98]. These computational models typically do not account for the mechanism of immune evasion and viral resistance, which significantly dictate molecular efficacy [98]. Additionally, they often overlook critical physiological variables that frequently coexist with HIV infection, such as gastrointestinal inflammatory conditions, which impair drug absorption; altered expression profiles of cytochrome P450 (CYP450) isoenzymes, which modulate drug metabolism; and other systemic factors that perturb drug distribution [98, 99].

## 8 Future Perspectives

Recent studies have demonstrated significant advances in molecular structural optimization of thiazole and thiazolidine derivatives in the field of efficacy and safety of new antivirals against HIV‐1. Chemical modifications introducing electronegative groupments, heteroatoms, or lipophilic side chains are important strategies to increase affinity of molecules to the allosteric site of HIV‐1 RT, besides improvement of metabolic stability and bioavailability [100]. Therefore, these strategies represent a promising approach in the drug design of next generation NNRTIs of next generations against resistant strains [101].

The molecular hybridization directed to inhibit the virus in different steps of HIV‐1 replication represents a promising strategy for the development of new potent compounds, especially with the incorporation of thiazole and thiazolidine scaffolds [72, 102]. Notably, these multitarget compounds can inhibit RT and integrase enzymes that are essential for viral replication and integration of viral genetic information into host cells [103]. Furthermore, this strategy can decrease the probability of viral resistance.

The Structure‐Based Drug Design (SBDD) strategy has benefited the design of new NNRTIs significantly, exploring the availability of computational crystallographic data as example viral enzymes in molecular docking experiments, improving drug discovery. The SBDD approach on the design of new NNRTIs aims to optimize hydrophobic interactions, i.e. pi–pi stacking and hydrogen bonds, improving affinity and stability of new inhibitors [104, 105].

Furthermore, a highly promising future prospect lies in the application of a therapeutic strategy based on the combination of drugs with distinct mechanisms of action targeting both HIV and the host immune system, thereby facilitating synergy and the potentiation of pharmacological effects [106]. Currently, combination antiretroviral therapy (cART) is extensively employed, relying on the inhibition of multiple stages of the viral replication cycle, which results in a more effective control of HIV‐1 replication, transmission, infection, and dissemination [107]. In this context, novel molecules bearing thiazole and/or thiazolidine scaffolds may be incorporated into this therapeutic modality in the future, potentially leading to enhanced efficacy of antiretroviral therapy, particularly concerning drug‐resistant strains.

Notably, immunotherapy‐based studies have also demonstrated that, when combined with pharmacological treatment, this approach has increasingly emerged as an adjuvant therapy. This occurs due to its capacity to induce a significant enhancement in immune activity, particularly in antigen‐presenting cells (APCs), which, alongside antiretroviral therapy, may lead to superior control of the viral infection and potentially the complete eradication of the virus from immune reservoirs [108, 109, 110]. Therefore, building upon the promising in silico and in vitro results obtained for thiazole and thiazolidine derivatives, these classes of compounds could, in the future, be combined with immunotherapeutic strategies to ensure enhanced in vivo antiviral and immunomodulatory activities. However, to realize this potential, it is imperative that in vivo antiviral evaluation models for these compounds be rigorously validated, as the literature remains scarce regarding such studies.

Although the thiazole and thiazolidine derivatives have good potential in RT‐HIV inhibition, they are a challenge in clinical applications due to the pharmaceutical chain of approval of new medicines. Moreover, the complexity of HIV‐1 resistance mechanisms requires ongoing research into the design of new drugs. Furthermore, the development of new compounds needs a balance between efficacy and safety, as well as cost, in order to guarantee population accessibility and treatment compliance.

## 9 Conclusion

Thiazole, thiazolidine, and thiazolidine‐carbonylated derivatives have demonstrated promising scaffolds in the design of potential non‐nucleoside inhibitors of HIV‐1 RT. However, the current scientific literature remains limited regarding specific investigations on their application as prototypes for adjuvant therapies in the management of HIV‐1 infection. Most studies focus on their direct antiviral properties, leaving aside their potential applications in modulating the immune response or the synergistic potential with already established antiretrovirals. Therefore, further investigations are needed to elucidate the possible role of these compounds in adjuvant approaches, which may open new perspectives for integrated therapeutic strategies against HIV‐1.

## Funding

This work was supported by Coordenação de Aperfeiçoamento de Pessoal de Nível Superior; Fundação de Amparo à Ciência e Tecnologia do Estado de Pernambuco; Conseil National de la Recherche Scientifique.

## Conflicts of Interest

The authors declare no conflicts of interest.

## Data Availability

Data sharing not applicable to this article as no datasets were generated or analysed during the current study.
